# Analysis of the Ballistic Effectiveness of the Hybrid Composite of Polyurethane and Kevlar 29 with Different Grammages

**DOI:** 10.3390/polym17030372

**Published:** 2025-01-29

**Authors:** Daniel Francisco Leiva Palomera, María Elena Fernández Abreu, José Luis Valín Rivera, Meylí Valin Fernández, Wanderley Ferreira de Amorim Júnior, Francisco Rolando Valenzuela Diaz, Diego Alejandro Alcaino Molina, Pablo Esteban Mendez Jofre, Cristobal Ignacio Galleguillos Ketterer

**Affiliations:** 1 Escuela de Ingeniería Mecánica, Pontificia Universidad Católica de Valparaíso, Valparaíso 2340025, Chile; daniel.leiva.p@mail.pucv.cl (D.F.L.P.); maria.fernandez.a@pucv.cl (M.E.F.A.); diego.alcaino.m@mail.pucv.cl (D.A.A.M.); pablo.mendez.j@mail.pucv.cl (P.E.M.J.); cristobal.galleguillos@pucv.cl (C.I.G.K.); 2 Department of Mechanical Engineering (DIM), Faculty of Engineering (FI), University of Concepción, Concepción 4030000, Chile; mvalin@udec.cl; 3 Departamento de Engenharia Mecânica, Universidade Federal de Campina Grande (UFCG), Paraíba 58429-900, Brazil; wanderley.ferreira@professor.ufcg.edu.br; 4 Departamento de Engenharia Metalúrgica e de Materiais, Escola Politécnica, Universidade de São Paulo (USP), São Paulo 05508-220, Brazil; frrvdiaz@usp.br

**Keywords:** composite material, Kevlar, ballistics, lamination, thermoset, 9 mm FMJ

## Abstract

In this study, the ballistic effectiveness of Kevlar 29 composites was analyzed by combining 400 and 460 GSM grammages with a polyurethane matrix. Plates measuring 300 mm × 250 mm were fabricated using hand lamination and compression techniques, with reinforcement designs consisting of 10, 14, and 18 layers of Kevlar 29 fabric oriented at a 90° angle. Ballistic tests were conducted following the parameters of the NIJ 0108.01 standard, applying five impacts with 9 mm FMJ and 22 (5.5 mm) caliber bullets. Both the composites and pure Kevlar were evaluated. Post-damage visual analysis was conducted on the front and back faces, as well as the interior of the composite, to identify delamination and fractures. The results show that .22 caliber bullets were captured at various stacking levels depending on the configuration, whereas 9 mm bullets penetrated all the plates. Localized fractures and delamination associated with the impacts were observed, highlighting the importance of stacking design and grammage in the material’s energy dissipation capacity.

## 1. Introduction

The research and development of ballistic armor has advanced significantly in recent years, focusing on improving materials to provide protection without compromising the mobility or comfort of users [1]. Thermoset polymers, such as polyurethane, have emerged as key components in the manufacture of composite materials due to their flexibility and impact-absorption capacity [2]. When combined with high-strength fibers such as Kevlar, hybrid composites are created that enhance the individual properties of each material [3].

Kevlar is recognized for its high stiffness and tensile strength, making it ideal for manufacturing protective vests against low-caliber weapons [4]. However, Kevlar fibers exhibit low adhesion with most polymeric matrices, resulting in reduced interlaminar shear strength [5]. On the other hand, polyurethane resin offers good chemical compatibility and excellent adhesion to various surfaces, making it a suitable matrix for Kevlar composites [6].

Recent studies have explored the incorporation of flexible polymeric matrices to improve the energy absorption capacity of composite materials [7]. However, the need to optimize the interaction between reinforcing fibers and the polymeric matrix persists to increase impact resistance and reduce interlaminar failures [8]. The integration of polyurethane as a matrix in composites reinforced with Kevlar fibers has proven to be an effective solution for improving stress distribution and energy absorption during ballistic impacts [9,10].

Hybrid materials, which combine two or more different materials to leverage the unique properties of each, such as strength, stiffness, ductility, and impact resistance, were the focus of this study. This work examines hybrid composites of polyurethane and Kevlar, evaluating their ballistic performance according to the NIJ 0108.01 standard [11]. The novelty of this study lies in the assessment of two specific weights of Kevlar combined with a flexible resin and their impact on the ballistic properties of the final composite, an approach that has been scarcely explored in previous research. For this purpose, plates were manufactured using hand lay-up and compression processes, utilizing Kevlar fabrics of different weights. The ballistic tests revealed failures, such as interface delamination, fiber fractures, and matrix degradation, under controlled impact conditions [12,13].

The implementation of flexible polyurethane matrices in these composites could significantly enhance ballistic impact absorption, which would have important implications not only in the design of personal protective equipment but also in military over-reinforcement applications or industrial applications where particle projections occur [14,15]. Improving energy absorption and impact resistance not only increases user safety but also extends the equipment’s service life [16,17]. A detailed understanding of the mechanical and ballistic behaviors of polyurethane and Kevlar composites is essential for their successful application in the ballistic field [18,19,20].

## 2. Materials and Methods

### 2.1. Materials

For the fabrication of the hybrid composites, a hand lay-up molding method with compression was employed, using the following materials and tools:

- Aramid: Kevlar 29 was used, woven in a 3000 Denier plain weave, corresponding to a ballistic fabric. Two different weights of Kevlar 29 were employed—400 GSM and 460 GSM—serving as reinforcement. The Kevlar 29 fabric was supplied by DuPont (Wilmington, DE, USA). Their properties are shown in Table 1.
- Biresin U1404, a polyurethane resin from the SIKA brand (Baar, Switzerland), was used as the base matrix, and was selected for its flexibility, adhesive capabilities, and ease of handling and cleaning. Its properties are shown in Table 2. Additionally, this resin significantly enhances impact resistance by helping to absorb and dissipate impact energy, reducing the likelihood of damage or penetration to the composite. It also improves dimensional stability, maintaining the shape and structure of the composite, even under high-temperature or high-humidity conditions. The polyurethane resin increases adhesion between the Kevlar fibers and the matrix, enhancing the composite’s tensile and impact resistance. Finally, it increases durability by protecting the Kevlar fibers from degradation and wear, extending the composite’s service life.
- Liquid Wax-815 Release Agent by SIKA (Baar, Switzerland), which facilitates the removal of the composite from the mold, is compatible with steel molds and is specifically designed for polyurethane resins.

#### Tools and Equipment

The following tools and equipment were essential for the preparation and fabrication of the composites:

Pinking Shears, which were used to cut Kevlar layers into the required dimensions. These shears are specifically designed for woven fibers, ensuring precise cuts and preventing fiber fraying.

Lamination Rollers, which were employed to ensure uniform resin impregnation in the Kevlar layers during the hand lay-up process, while also eliminating interlaminar air bubbles.

Compression Molds, which were constructed from A36 steel with internal dimensions of 300 mm × 250 mm. These molds were fabricated at the School of Mechanical Engineering of PUCV. Designed by the author, they were used to maintain the stacking configuration during the fabrication and curing process under pressure.

Compression Press, which is a hydraulic press used to compact and cure the composite in the mold under a pressure of 80 bar at an ambient temperature of 25 to 28 °C.

### 2.2. Calculation of the Volume Fraction

The volume fraction of a component is defined as the ratio between the volume of reinforcement and the total volume of the composite material. It can be mathematically expressed according to Equation (1) [21], as follows:

(1) $$v_{f}=\frac{\frac{\omega_{j}}{x_{j}}}{\frac{\omega_{j}}{x_{j}}+\frac{\omega_{m}}{x_{m}}}[\%]$$

where $v_{f}$ represents the volume fraction of the fibers in the laminate, *ω_j_* and *ω_m_* denote the weights of the Kevlar fibers and the matrix, respectively, and *x_j_* and *x_m_* correspond to the densities of the Kevlar fibers and the matrix, which are 1.44 [g/cm^3^] and 1.05 [g/cm³], respectively.

### 2.3. Calculation of Surface Density

The surface density in armor refers to the amount of mass per unit area distributed over the surface of the composite material [16]. To calculate the surface density (σ*)*, Equation (2) was applied, as follows:

(2) σ=mA[kgm2]

### 2.4. Fabrication

#### 2.4.1. Fabrication of Compression Molds

**Figure 1:**
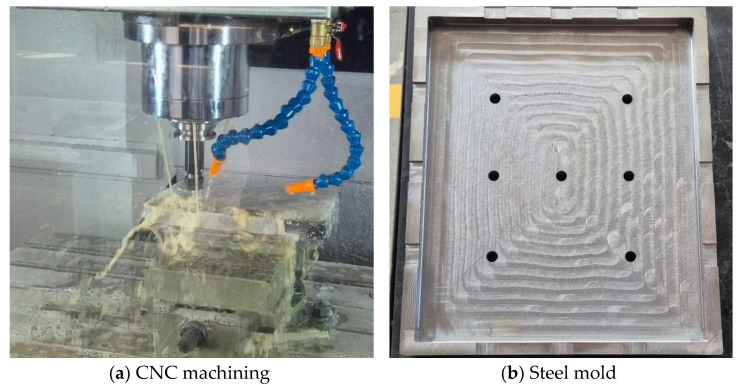
Steel mold fabrication.

Before proceeding with the fabrication of the composites, a mold with dimensions of 300 mm × 250 mm was designed, referencing a commercially available ballistic plate of size M used in bulletproof vests. The mold was manufactured from ASTM A36 steel using CNC equipment at the Mechanical Engineering School of the Pontificia Universidad Católica de Valparaíso. Figure 1a shows the milling process of the mold’s interior and Figure 1b shows the mold after CNC milling. The visible holes are threaded (M12) to facilitate demolding of the composite.

#### 2.4.2. Preparation of Materials

The Kevlar 29 fabric was cut into dimensions of 300 mm × 250 mm using pinking shears, ensuring precision in the cuts and preventing fiber fraying.

The polyurethane resin, Biresin U1404, was prepared according to the manufacturer’s specifications. The components were mixed in the appropriate proportions to ensure proper polymerization and optimal mechanical properties.

#### 2.4.3. Stacking Design

The Kevlar layers were arranged in a stacking sequence by group, as shown in Table 3. The first three layers were 400 GSM, followed by subsequent layers of 460 GSM. In Plate 1, only the reinforcement material was used, without the Biresin matrix. The design of the layer arrangement aimed to enhance ballistic impact absorption.

#### 2.4.4. Hand Lay-Up Lamination

The composite fabrication was performed using a hand lay-up lamination process in a steel mold. A first layer of polyurethane resin was applied to the mold, followed by the placement of Kevlar layers. Polyurethane resin was uniformly added between the layers using a flat spatula and a bubble roller. This process ensured that the resin fully impregnated the Kevlar fibers, minimizing the formation of air bubbles or areas without proper contact.

#### 2.4.5. Compression Molding

The composite was fabricated in an A36 steel mold. The compression process was performed using a press under a pressure of 80 bar, maintained for 24 h at an ambient temperature of 25 to 28 °C to ensure the desired properties were achieved.

### 2.5. Ballistic Test

#### 2.5.1. Fabrication of the Ballistic Plate Carrier

**Figure 2:**
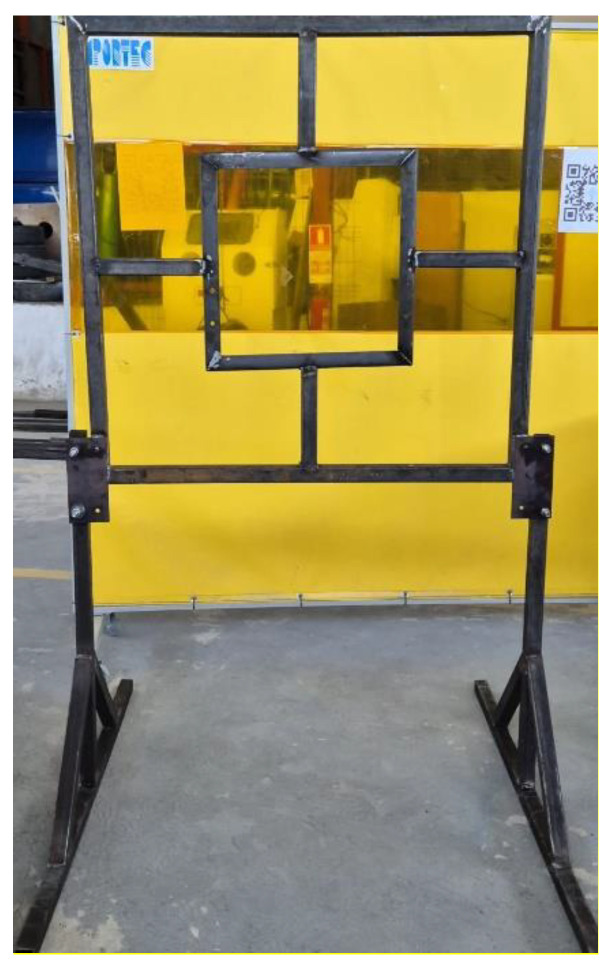
Fabricated plate carrier.

To conduct the ballistic tests, it was necessary to design and fabricate a plate holder with dimensions of 2 m in height and 1.3 m in width. Figure 2 shows the completed holder. This plate holder ensured the proper fixation of samples measuring 300 mm × 250 mm and was adaptable to sizes up to a maximum of 500 mm × 500 mm. Designed and built by the author, this device guaranteed the necessary strength and stability to meet the objectives of the tests.

#### 2.5.2. Ballistic Impact Test

**Figure 3:**
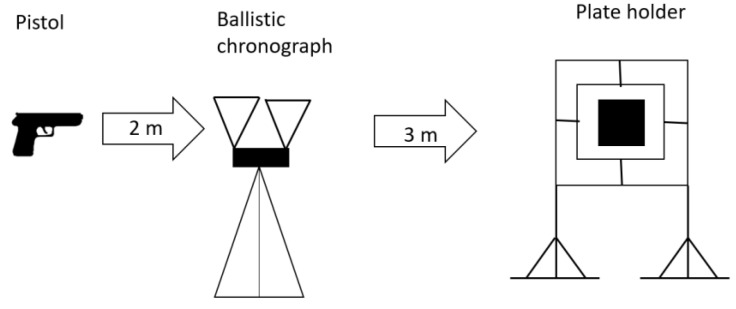
Sketch of the ballistic test setup.

The fabricated samples were subjected to ballistic tests following the parameters established in the NIJ0108.01 [22] standard, using the ammunition calibers specified in Table 4. For level I armor, .22 caliber ammunition was used, fired from a Smith & Wesson Victory pistol, manufactured by Smith & Wesson in Springfield, Massachusetts, United States, at a velocity of 330 m/s, measured with a ballistic chronograph positioned 2 m from the firearm’s muzzle to minimize measurement errors caused by expelled gases and gunpowder. For level IIIA armor, 9 mm caliber ammunition was used, fired from a Mauser mod. 90DA pistol, manufactured by Mauser in Oberndorf am Neckar, Germany, at a velocity of 440 m/s. The plates were impacted at a distance of 5 m for both calibers, measured from the tip of the firearm barrel, as shown in Figure 3.

The objectives of these tests were to evaluate penetration and perform a macroscopic visual analysis of the deformation in the plates, in compliance with the requirements established in the NIJ 0108.01 standard [22]. This analysis provides key information about the composite material’s ability to absorb and distribute impact energy, allowing for validation of its performance and ensuring compliance with regulatory standards. The observed damage includes characteristics such as plastic deformation, permanent dents, fractures, or cracks around the impact area, depending on the surface density of the material and the energy transferred by the projectile. At the entry hole, clean or irregular edges can be identified, whereas the exit hole, if present, is generally larger and shows material displaced outward. Burn or friction marks may also be visible on the plate’s surface, as well as fragments from both the projectile and material detached from the plate.

### 2.6. Macrostructural Analysis

The macrostructural analysis was performed visually, examining each area affected by the impacts, evaluating the overall integrity of the material, and identifying its failure mechanisms and the type of fracture exhibited.

## 3. Results

### 3.1. Volume Fraction and Surface Density

The calculated values for the fiber volume fraction and surface density for each plate are presented in Table 5. Plates with a higher number of layers exhibited greater surface density. It is worth noting that Plate 1 consisted of pure Kevlar without resin and without a compaction process.

### 3.2. Ballistic Impact Results

Plate 1 was subjected to two ballistic impacts at a distance of 5 m from the barrel’s muzzle, the first with 5.5 mm caliber ammunition and the second with 9 mm caliber ammunition. In both cases, the impacts fully penetrated the plate, as indicated in Table 6. This result demonstrates that the plate lacks the structural capacity to withstand the impact of the aforementioned ammunition.

Plate 2 was subjected to four ballistic impacts at a distance of 5 m from the barrel’s muzzle, as detailed in Table 7. For impacts 1 and 2, 9 mm caliber ammunition was used, resulting in full penetration, indicating that the plate was unable to withstand the 772 J of energy generated by this projectile. However, in shots 3 and 4, using lighter 5.5 mm caliber ammunition, the plate successfully stopped the projectiles, demonstrating its resistance to impacts with energy lower than 134 J.

In the tests for Plates 3 and 4, ballistic impacts were conducted at a distance of 5 m from the barrel’s muzzle. Impacts 1 and 2, using 9 mm caliber ammunition, resulted in full penetration, indicating that the plates failed to withstand the 772 J of energy. However, in shots 3, 4, 5, and 6, using lighter 5.5 mm caliber ammunition, the plates successfully stopped the projectiles, as shown in Table 8 and Table 9. This indicates that both plates can withstand impacts with energy lower than 134 J.

### 3.3. Macroscopic Analysis of the Plates

#### 3.3.1. Macroscopic Analysis of Plate 1

**Figure 4:**
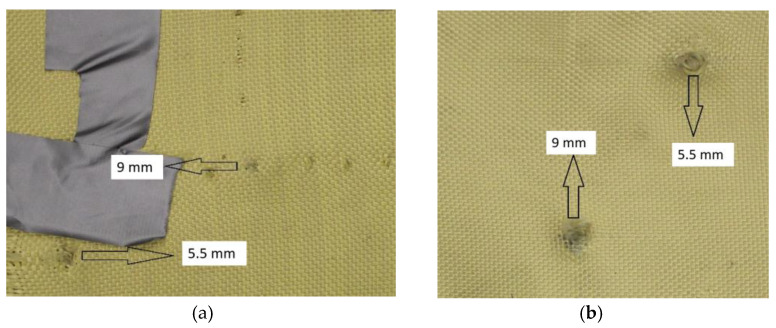
(**a**) Ballistic impacts on Plate 1 front side. (**b**) Ballistic impacts on Plate 1 rear side.

As shown in Figure 4, Plate 1 exhibited fiber unraveling in the area surrounding the impact point upon impact. 

#### 3.3.2. Macroscopic Analysis of Plate 2

**Figure 5:**
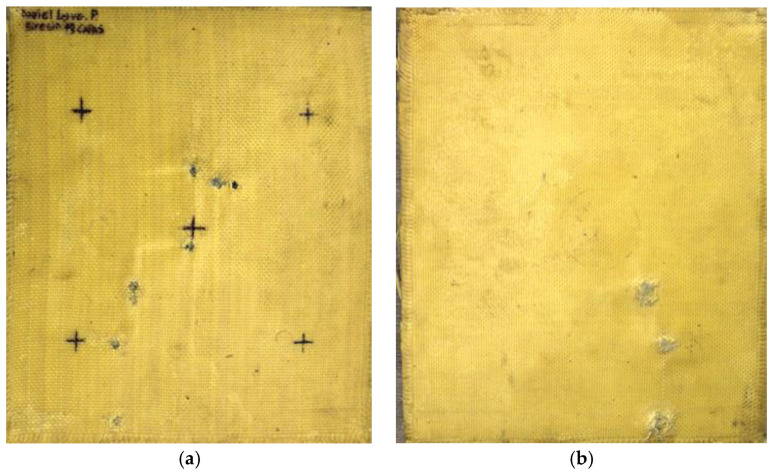
(**a**) Ballistic impacts on Plate 2 front side. (**b**) Ballistic impacts on Plate 2 rear side.

In Plate 2, the impacts caused by the 9 mm caliber projectile resulted in full penetration, unlike the impacts caused by the 5.5 mm caliber projectile, all of which were retained, as shown in Figure 5.

#### 3.3.3. Macroscopic Analysis of Plate 3

**Figure 6:**
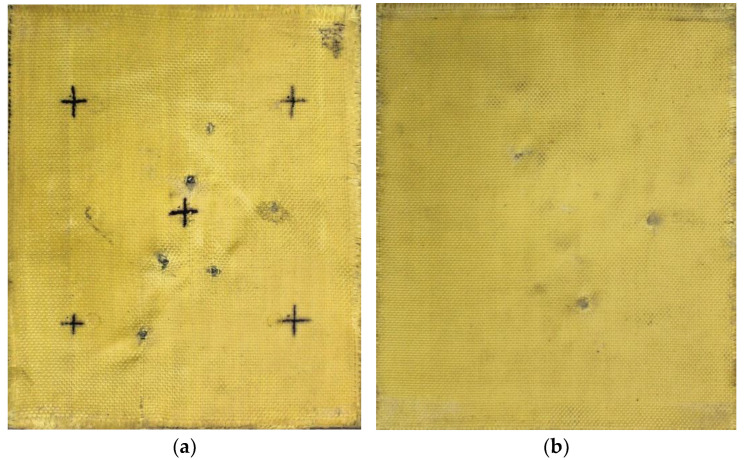
(**a**) Ballistic impacts on Plate 3 front side. (**b**) Ballistic impacts on Plate 3 rear side.

In Plate 3, the impacts caused by the 9 mm caliber projectile resulted in full penetration, unlike the impacts caused by the 5.5 mm caliber projectile, all of which were retained, as shown in Figure 6.

#### 3.3.4. Macroscopic Analysis of Plate 4

**Figure 7:**
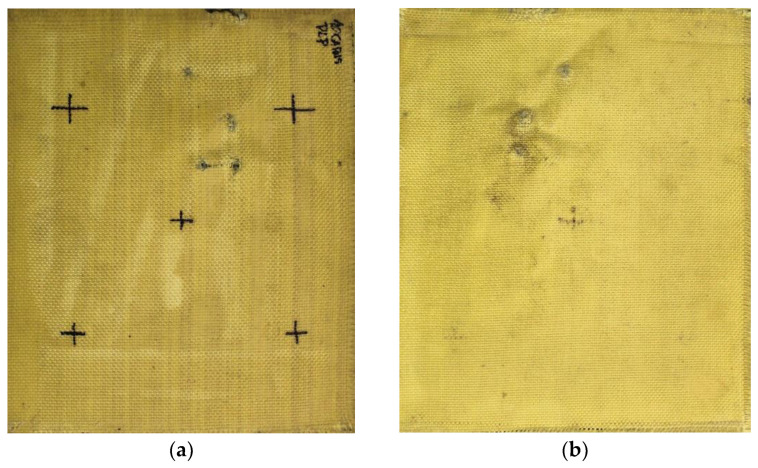
(**a**) Ballistic impacts on Plate 4 front side. (**b**) Ballistic impacts on Plate 4 rear side.

In Plate 4, the impacts caused by the 9 mm caliber projectile resulted in full penetration, unlike the impacts caused by the 5.5 mm caliber projectile, all of which were retained, as shown in Figure 7.

The macroscopic analysis demonstrates the poor performance of Plate 1 against impacts from both 9 mm and 5.5 mm projectiles.

This analysis also highlights the excellent performance of Plates 2, 3, and 4 against 5.5 mm projectile impacts; however, they did not exhibit the same behavior when subjected to 9 mm projectile impacts.

### 3.4. Macroscopic Analysis of Projectile Trajectories

For the macroscopic analysis of the projectile trajectories, each layer of the material was separated.

#### 3.4.1. Analysis of Bullet Trajectories in Plate 1

According to the grammages used (400 GSM and 460 GSM), the deformation was analyzed at the transition from one grammage to the other and for each plate variant, specifically in layer 10, layer 14, and layer 18.

**Figure 8:**
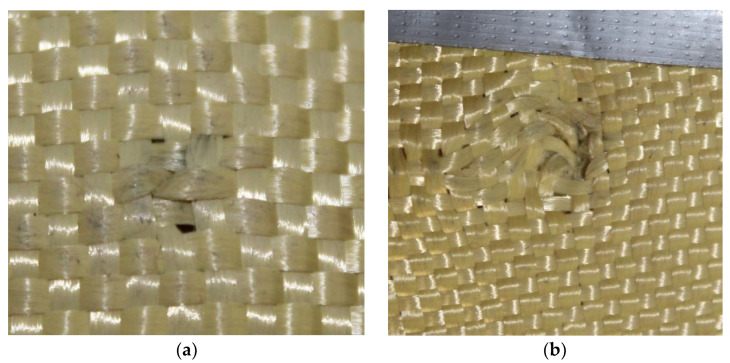
(**a**) Impact of 9 mm on Plate 1. (**b**) 5.5 mm projectiles on Plate 1.

In Figure 8a, the impact of the 9 mm projectile can be observed, highlighting a change in coloration as it perforates the plate. This corresponds to particles from the projectile due to its high impact velocity, which generates elevated friction and disrupts the alignment of the Kevlar weave.

In Figure 8b, the impact of the 5.5 mm projectile is visible, also showing a change in coloration as it perforates the plate. This was attributed to particles from the projectile, with its high velocity causing elevated friction and a greater disruption in the Kevlar weave’s orientation compared to the 9 mm caliber projectile.

**Figure 9:**
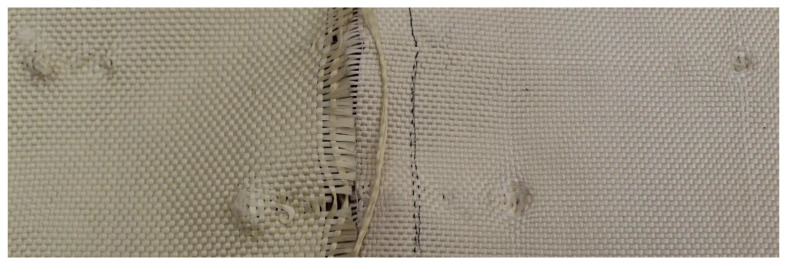
Transition from 400 to 460 GSM grammage.

When analyzing the samples with different grammages, the 460 GSM grammage exhibited less deformation compared to the 400 GSM grammage, as shown in Figure 9.

**Figure 10:**
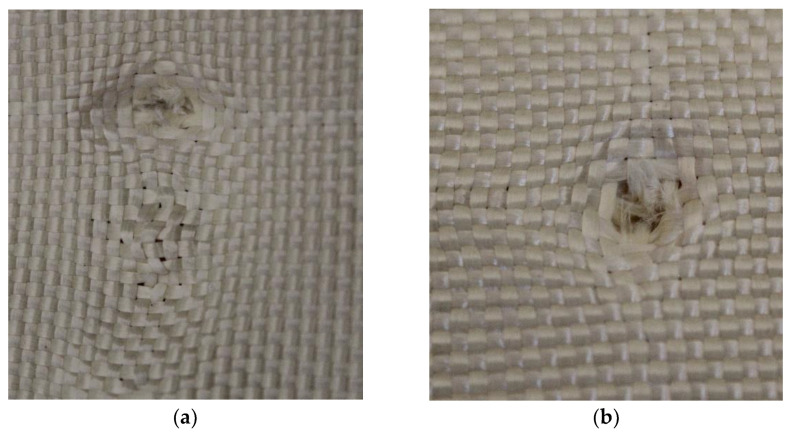
(**a**) entry of the 9 mm projectile in layer 10 of Plate 1. (**b**) exit of the 9 mm projectile in layer 10 of Plate 1.

**Figure 11:**
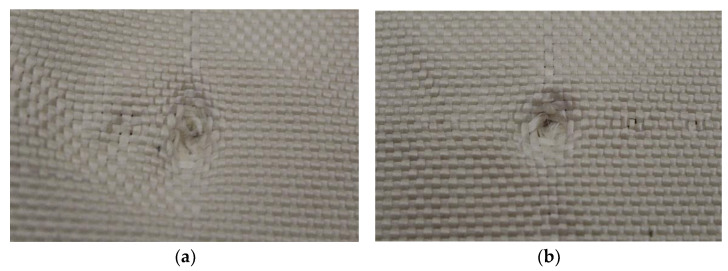
(**a**) entry of the 9 mm projectile in layer 14 of Plate 1. (**b**) exit of the 9 mm projectile in layer 14 of Plate 1.

Figure 10 and Figure 11 show the trajectories of the 5.5 mm and 9 mm projectiles, respectively, starting from layer 10. The 9 mm projectile created a uniform deformation zone, unlike the 5.5 mm projectile. The same observation applies to the analysis of the trajectories of both projectiles, starting from layer 14.

**Figure 12:**
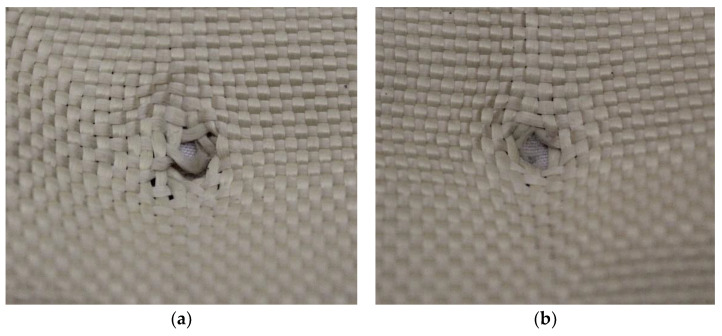
(**a**) entry of the 9 mm projectile in layer 18 of Plate 1. (**b**) exit of the 9 mm projectile in layer 18 of Plate 1.

The analysis of layer 18, as shown in Figure 12, reveals the exit hole created by both projectiles. The observed damage includes conical cavitation, which indicates the high-energy impact caused by the projectile, intra-laminar shear resulting from the layers sliding against each other under stress, and significant plastic deformation. These findings provide insight into the failure mechanisms of the composite under ballistic impact conditions.

#### 3.4.2. Analysis of Bullet Trajectories in Plate 2

**Figure 13:**
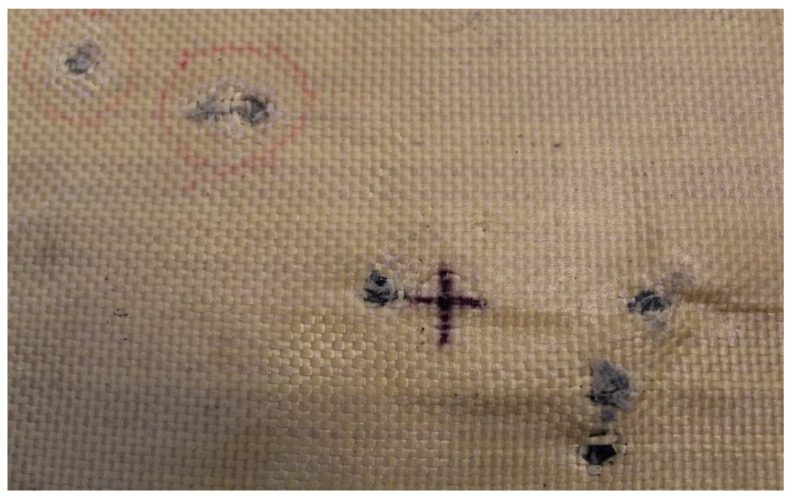
Ballistic impacts on Plate 2.

Analysis of the projectile impacts demonstrated the following: the 9 mm projectile fully penetrated, whereas the 5.5 mm projectile did not. The 9 mm projectile did not cause apparent deformation at the entry hole, whereas the 5.5 mm projectile created superior conical widening. For both calibers, material rupture and discoloration around the impact area were observed, as shown in Figure 13.

**Figure 14:**
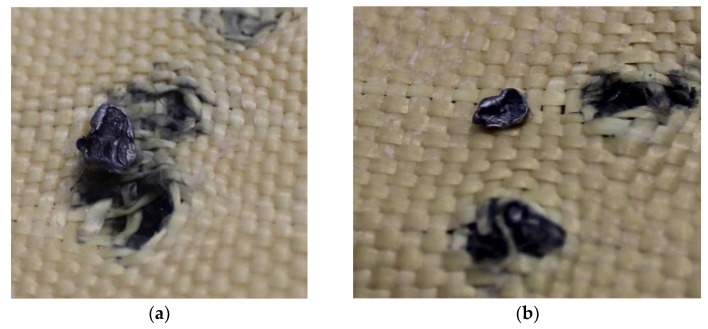
(**a**) Projectile fragments in layer 2 on Plate 2. (**b**) Projectile fragments in layer 5 on Plate 2.

The first fragments of 5.5 mm projectiles were found in layer 2, as shown in Figure 14a. The analysis also revealed a bullet fragment in layer 5, as depicted in Figure 14b.

**Figure 15:**
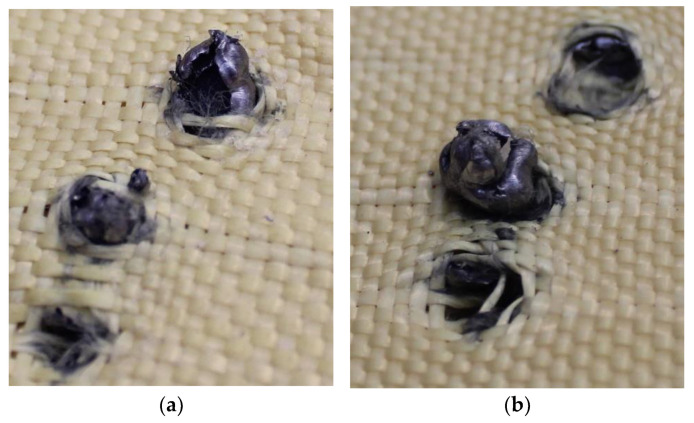
(**a**) Projectile fragments in layer 2 on Plate 2. (**b**) Projectile fragments in layer 5 on Plate 2.

Figure 15a,b shows the fragments of 5.5 mm projectiles retained in layers 3 and 4, highlighting the deformation of the projectiles, as well as deterioration in the Kevlar-affected areas caused by the deposition of projectile particles.

**Figure 16:**
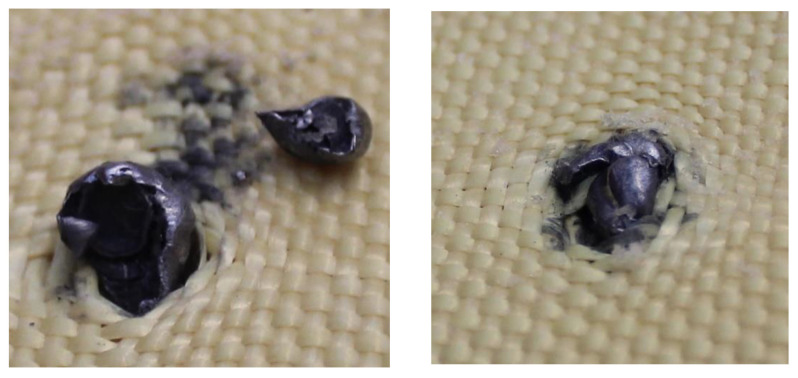
(**a**) Projectile fragments in layer 5 on Plate 2. (**b**) Projectile fragments in layer 6 on Plate 2.

Figure 16a,b show the fragments of 5.5 mm projectiles retained in layers 5 and 6, highlighting deformation of the projectiles and the material deposited in the composite as a result of this deformation.

#### 3.4.3. Analysis of Projectile Trajectories in Plate 3

**Figure 17:**
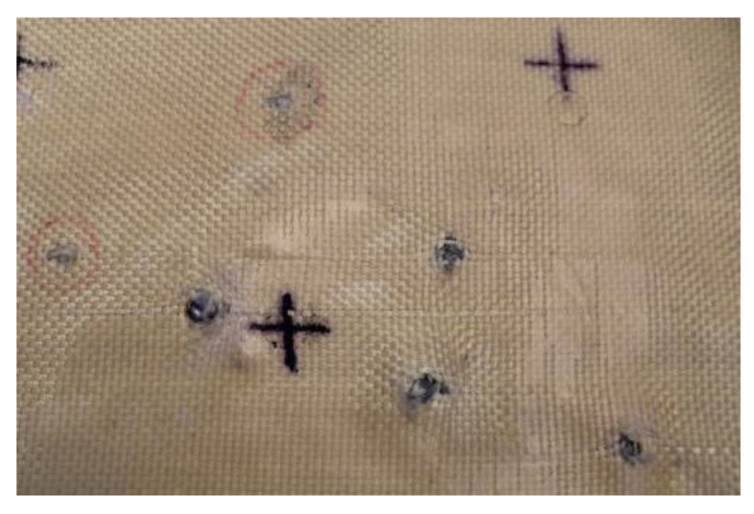
Ballistic impacts on Plate 3.

Analysis of the projectile impacts demonstrated the following: the 9 mm projectile fully penetrated, whereas the 5.5 mm projectile did not. The 9 mm projectile did not cause apparent deformation at the entry hole, whereas the 5.5 mm projectile created superior conical widening. For both calibers, material rupture and discoloration around the impact area were observed, as shown in Figure 17.

**Figure 18:**
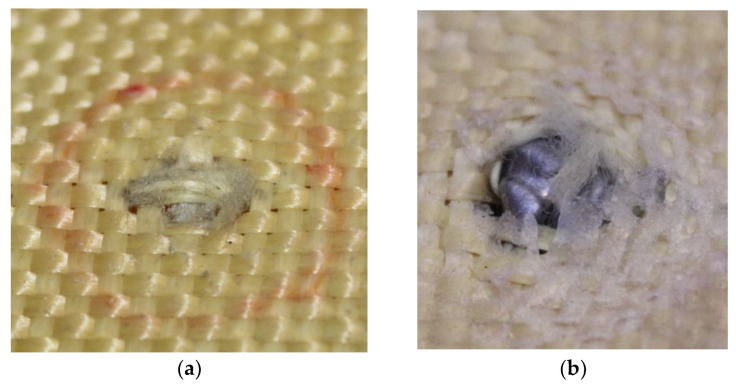
(a) 9 mm projectile entry point and (b) 5.5 mm projectile entry point.

In Figure 18a, the entry point of the 9 mm projectile is observed, whereas in Figure 18b, the entry point of the 5.5 mm projectile is shown, with the latter protruding from the first layer.

**Figure 19:**
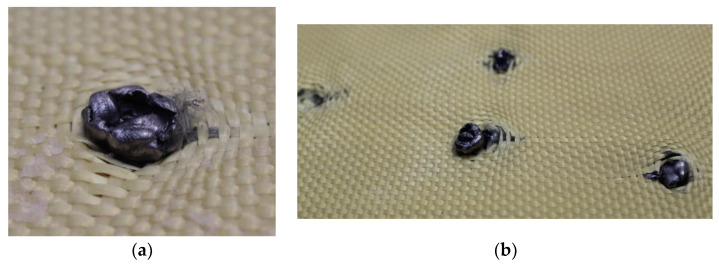
(**a**) Projectile fragments in layer 2 on Plate 3. (**b**) Projectile fragments in layer 3 on Plate 3.

In Figure 19a, a fragment of the 5.5 mm projectile was retained in layer 2, whereas in Figure 19b, multiple fragments of the same caliber were detected, which were retained in layer 3.

**Figure 20:**
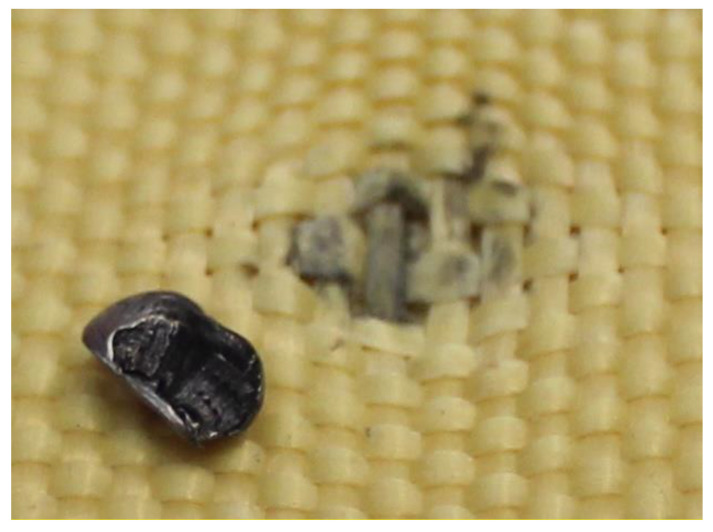
Fragment of the 5.5 mm projectile in layer 5.

Although most fragments were retained in layer 3, one fragment was retained in layer 5, as shown in Figure 20, causing damage to the material.

#### 3.4.4. Analysis of Projectile Trajectory in Plate 4

**Figure 21:**
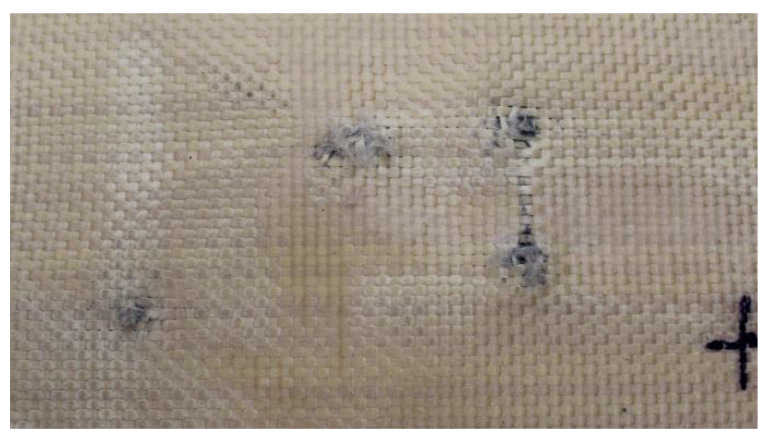
Ballistic impacts on Plate 4.

In Plate 4, which had the smallest stacking, a behavior similar to that of the previously analyzed plates was observed, as shown in Figure 21.

**Figure 22:**
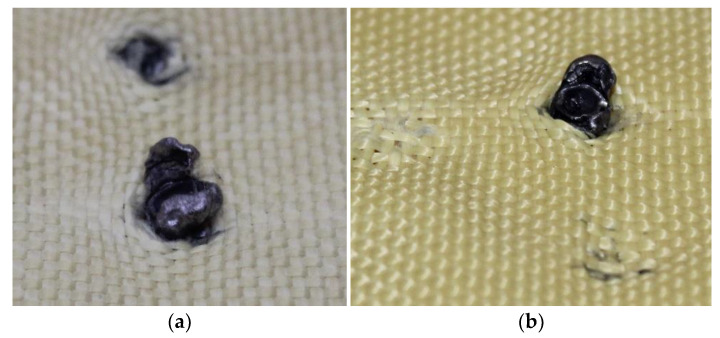
(**a**) Projectile fragments in layer 4 on Plate 4. (**b**) Projectile fragments in layer 6 on Plate 4.

**Figure 23:**
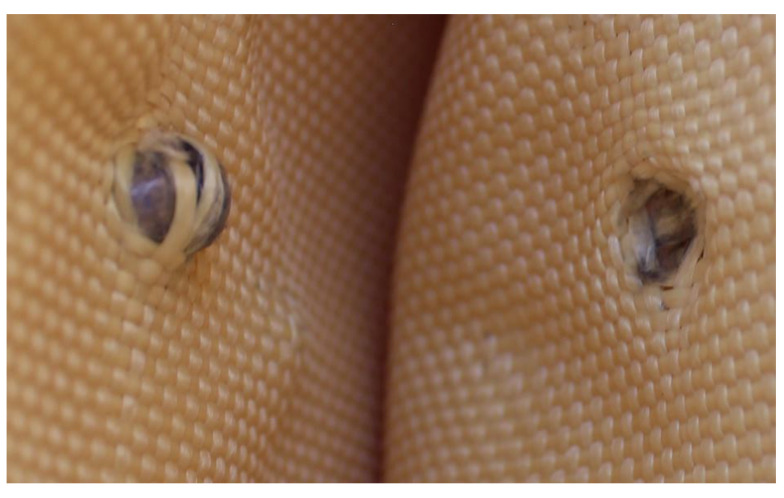
Fragment of the 5.5 mm projectile in layer 6.

The 5.5 mm projectiles were retained in layers 4 and 6, as shown in Figure 22 and Figure 23, respectively. Significant deformation was observed at the impact zones where the projectiles were retained. Additionally, detachment of the composite material was evident; however, the Kevlar weave successfully retained the projectiles.

### 3.5. Analysis of Retained Projectiles

**Figure 24:**
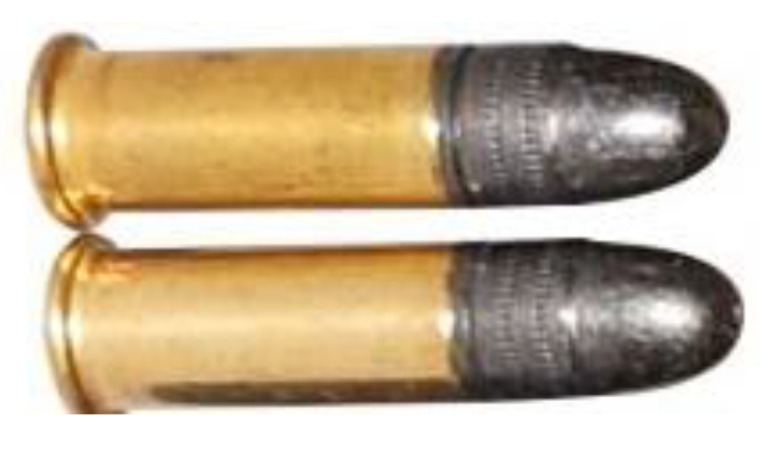
.22 caliber bullets.

**Figure 25:**
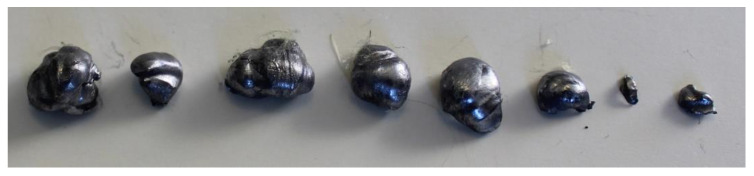
.22 caliber bullets in Plate 2.

**Figure 26:**
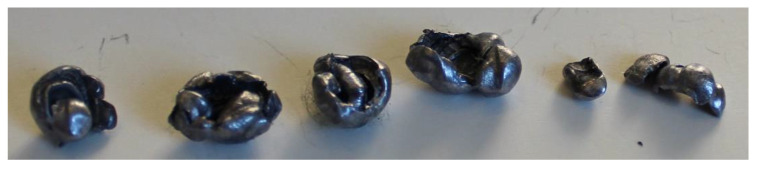
.22 caliber bullets in Plate 3.

**Figure 27:**
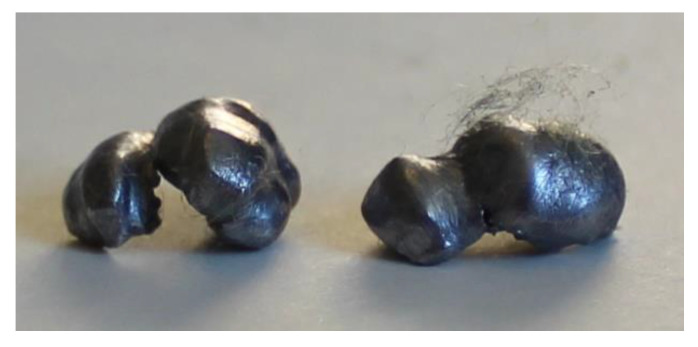
.22 caliber bullets in Plate 4.

The analysis of the projectiles extracted from Plates 2, 3, and 4 reveals that they exhibit similar shapes depending on the plate from which they were recovered. Notably, as the surface density of the composite increases, the projectiles experience greater fragmentation. This is evident when comparing Figure 24, which shows an unfired .22 caliber projectile, to Figure 25, Figure 26 and Figure 27, which display the fragments recovered from the plates. These fragments reveal significant plastic deformation and rupture, resulting in a greater number of fragments, except in the case of Plate 4 (Figure 27), where the projectiles did not fragment.

This analysis provides critical data on the behavior of the composite material under impact, enabling an assessment of its energy absorption and distribution capacities, which are essential aspects of its mechanical performance. Additionally, it aids in identifying potential structural weaknesses in the composite, contributing to the design of improvements that enhance its resistance and effectiveness in future applications.

## 4. Conclusions

The results indicate that plates with a higher fiber volume fraction exhibit greater surface density. However, none of the composite plates were able to stop impacts from 9 mm caliber projectiles, although they did successfully retain 5.5 mm caliber projectiles, except for Plate 1, which was made of pure Kevlar and showed no resistance to the tested projectiles. This suggests that although increasing the fiber content enhances ballistic resistance against smaller calibers, it is insufficient to withstand higher calibers, such as 9 mm calibers. Factors such as fiber distribution, adhesion between the matrix and reinforcement, and the projectile’s kinetic energy must be considered in future studies.

The results obtained in this study reveal the ballistic behavior of hybrid composites made of polyurethane and Kevlar 29 with different grammages. It was found that incorporating polyurethane resin as a matrix significantly improved the composite material’s ability to resist low-energy projectile impacts (138 J) compared to the pure Kevlar plate. This confirms that the matrix plays a crucial role in structural cohesion and stress distribution, increasing energy absorption. Furthermore, composites with higher surface density demonstrated better performance against ballistic impacts.

The analysis of ballistic test images shows that Kevlar with 460 GSM exhibited the best capacity for energy absorption and impact stress distribution.

## Figures and Tables

**Table 1:** Properties of Kevlar.

Properties	Kevlar 29
Density [gcm3]	1.44
Young’s Modulus [GPa]	83
Tensile Strength [GPa]	3.6
Elongation at Break [%]	4

**Table 2:** Properties of Biresin Resin.

Properties	Biresin U1404
Density [gcm3]	1.05
Shore Hardness	A 40
Tear Resistance [GPa]	7
Tensile Strength [GPa]	3.0–4.0
Elongation at Break [%]	600
Color	Reddish–Transparent

**Table 3:** Stacking Design.

Plate	Total Number of Layers	Layer Distribution	Observations
Plate 1	18	3 layers of 400 GSM + 15 layers of 460 GSM	Reinforcement material only, without Biresin base matrix
Plate 2	18	3 layers of 400 GSM + 15 layers of 460 GSM	Includes Biresin base matrix
Plate 3	14	3 layers of 400 GSM + 11 layers of 460 GSM	Includes Biresin base matrix
Plate 4	10	3 layers of 400 GSM + 7 layers of 460 GSM	Includes Biresin base matrix

**Table 4:** Armor levels according to the NIJ 0108.01 standard.

Armor Level	Ammunition Type	Nominal Mass	Caliber Length	Reference Velocity	Shots per Plate
I	22 LRHV Lead	2.6 g/40 gr	15–16.5 cm	320 ± 12 m/s	5
	38 Special RN Lead	10.2 g/158 gr	15–16.5 cm	259 ± 15 m/s	
IIA	9 mm FMJ	8.0 g/124 gr	10–12 cm	332 ± 12 m/s	5
	357 Mag JSP	10.2 g/158 gr	10–12 cm	381 ± 15 m/s	
II	9 mm FMJ	8.0 g/124 gr	10–12 cm	358 ± 12 m/s	5
	357 Mag JSP	10.2 g/158 gr	15–16.5 cm	425 ± 15 m/s	
IIIA	9 mm FMJ	8.0 g/124 gr	24–26 cm	426 ± 15 m/s	5
	44 Mag Lead SWC Gas Checked	15.88 g/240 gr	14–16 cm	426 ± 15 m/s	
III	7.62 mm 308	9.7 g/150 gr	56 cm	838 ± 15 m/s	5
	Winchester FMJ				
IV	30–06 AP	10.8 g/166 gr	56 cm	868 ± 15 m/s	5

**Table 5:** Characteristics of the plates.

Characteristics of the Plates										
Plate	Number of Layers	Composite Thickness [mm]	Surface Area [m^2^]	Weight [kg]	Surface Density [kgm2]	Wm [gr]	Pm	Wj [gr]	Pj	Volume Fraction [%]
1	18	8.1	0.075	0.583	7.77	0	0	48.5	1.44	100
2	18	11	0.075	0.824	10.99	240	1.051	583	1.44	63.94
3	14	8.8	0.075	0.627	8.36	179	1.051	449	1.44	64.67
4	10	5.7	0.075	0.4	5.33	90	1.051	310	1.44	71.54

**Table 6:** Results for Plate 1.

Plate 1					
N°	Caliber	Projectile Energy [gr]	Velocity [m/s]	Penetration	Energy [J]
1	Fiocchi 9 × 19 FMJ luger	123	440	yes	772
2	5.5	38	330	yes	134

**Table 7:** Results for Plate 2.

Plate 2					
N°	Caliber	Projectile Energy [gr]	Velocity [m/s]	Penetration	Energy [J]
1	Fiocchi 9 × 19 FMJ luger	123	440	yes	772
2	Fiocchi 9 × 19 FMJ luger	123	440	yes	772
3	5.5	38	330	No	134
4	5.5	38	330	No	134

**Table 8:** Results for Plate 3.

Plate 3					
N°	Caliber	Projectile Energy [gr]	Velocity [m/s]	Penetration	Energy [J]
1	Fiocchi 9 × 19 FMJ luger	123	440	yes	772
2	Fiocchi 9 × 19 FMJ luger	123	440	yes	772
3	5.5	38	330	No	134
4	5.5	38	330	No	134
5	5.5	38	330	No	134
6	5.5	38	330	No	134

**Table 9:** Results for Plate 4.

Plate 4					
N°	Caliber	Projectile Energy [gr]	Velocity [m/s]	Penetration	Energy [J]
1	Fiocchi 9 × 19 FMJ luger	123	440	yes	772
2	Fiocchi 9 × 19 FMJ luger	123	440	yes	772
3	5.5	38	330	No	134
4	5.5	38	330	No	134
5	5.5	38	330	No	134
6	5.5	38	330	No	134

## Data Availability

The original contributions presented in this study are included in the article. Further inquiries can be directed to the corresponding authors.
